# Characterizing the fitness cost of viral escape from the HIV-1 broadly neutralizing monoclonal antibody VRC01

**DOI:** 10.1186/1742-4690-9-S2-P87

**Published:** 2012-09-13

**Authors:** RM Lynch, L Tran, X Wu, Y Li, B Lee, J Mascola

**Affiliations:** 1 NIH/NIAID, Bethesda, MD, USA; 2 The Scripps Research Institute, La Jolla, CA, USA; 3 UCLA School of Medicine, Los Angeles, CA, USA

## Background

The receptor-binding site on the HIV glycoprotein gp120 is a highly conserved epitope, and certain antibodies directed against this CD4 binding site (CD4bs) can potently neutralize the majority of circulating HIV-1 isolates. One such antibody, VRC01, was isolated from a slow progressor HIV-1 infected donor who maintained low to moderate viral load without treatment. We recently described that almost all viruses in this donor plasma had escaped VRC01 neutralization. This raised the question of whether viral escape from a broadly reactive CD4bs antibody results in reduced affinity for CD4 and thus, a fitness cost to viral replication.

## Methods

Env-pseudoviruses and infectious molecular clones (IMC) were constructed using near-full length gp160 env genes from three circulating VRC01-resistant viruses and their complementary revertants (where VRC01-sensitivity was restored through mutations in the CD4 binding loop, Loop D and V5) as well as from autologous env genes from the VRC01 donor (both sensitive and resistant to VRC01 neutralization). Cell entry was quantified by infectivity into cell-lines expressing varying levels of the CD4 receptor, and replication kinetics of IMC were assessed by in vitro infection of primary CD4 T cells.

## Results

Two of the three reverted VRC01-sensitive viruses demonstrated more efficient CD4 receptor mediated entry and greater replication in CD4 T cells, than the parental VRC01-resistant Envs. However, analysis of five VRC01-resistant and four VRC01-sensitive autologous Envs from the VRC01 donor revealed no significant difference in replication kinetics or efficiency of CD4 usage in infectivity assays.

## Conclusion

Some VRC01-resistant viruses appear to have impaired replicative fitness, possibly caused by reduced CD4-mediated cell entry. However, VRC01-resistant Envs derived from the VRC01 donor did not display this deficiency, suggesting that compensatory changes over time may partially or fully restore CD4 usage and replication.

